# Integrating Stand and Soil Properties to Understand Foliar Nutrient Dynamics during Forest Succession Following Slash-and-Burn Agriculture in the Bolivian Amazon

**DOI:** 10.1371/journal.pone.0086042

**Published:** 2014-02-07

**Authors:** Eben N. Broadbent, Angélica M. Almeyda Zambrano, Gregory P. Asner, Marlene Soriano, Christopher B. Field, Harrison Ramos de Souza, Marielos Peña-Claros, Rachel I. Adams, Rodolfo Dirzo, Larry Giles

**Affiliations:** 1 Department of Global Ecology, Carnegie Institution for Science, Stanford, California, United States of America; 2 Department of Biology, Stanford University, Stanford, California, United States of America; 3 Sustainability Science Program, Kennedy School of Government, Harvard University, Cambridge, Massachusetts, United States of America; 4 Department of Anthropology, Stanford University, Stanford, California, United States of America; 5 Instituto Boliviano de Investigación Forestal, Santa Cruz de la Sierra, Bolivia; 6 Centro de Investigación y Preservación de la Amazonia, Universidad Amazónica de Pando, Cobija, Bolivia; 7 Forest Ecology and Forest Management Group, Wageningen University, Wageningen, the Netherlands; 8 Plant and Microbial Biology, University of California, Berkeley, California, United States of America; University of Illinois, United States of America

## Abstract

Secondary forests cover large areas of the tropics and play an important role in the global carbon cycle. During secondary forest succession, simultaneous changes occur among stand structural attributes, soil properties, and species composition. Most studies classify tree species into categories based on their regeneration requirements. We use a high-resolution secondary forest chronosequence to assign trees to a continuous gradient in species successional status assigned according to their distribution across the chronosequence. Species successional status, not stand age or differences in stand structure or soil properties, was found to be the best predictor of leaf trait variation. Foliar δ^13^C had a significant positive relationship with species successional status, indicating changes in foliar physiology related to growth and competitive strategy, but was not correlated with stand age, whereas soil δ^13^C dynamics were largely constrained by plant species composition. Foliar δ^15^N had a significant negative correlation with both stand age and species successional status, – most likely resulting from a large initial biomass-burning enrichment in soil ^15^N and ^13^C and not closure of the nitrogen cycle. Foliar %C was neither correlated with stand age nor species successional status but was found to display significant phylogenetic signal. Results from this study are relevant to understanding the dynamics of tree species growth and competition during forest succession and highlight possibilities of, and potentially confounding signals affecting, the utility of leaf traits to understand community and species dynamics during secondary forest succession.

## Introduction

Secondary forests cover a large and expanding portion of tropical forests worldwide [1]. These forests provide valuable ecosystem services [2], including biodiversity corridors and refugia [3], wildlife habitat, water filtration, and forest products [4]. In addition, carbon uptake by secondary forests is an important factor in greenhouse gas emissions [5], with 30% of deforested areas in the Brazilian Amazon having been abandoned and now in some stage of regrowth [6]. Forest regeneration following slash-burn agriculture is of particular importance, as this activity has resulted in 50% of annual deforestation and 25% of estimated net greenhouse gas emissions in Asia [7]. Due to their fast growth rates, these forests may help alleviate deforestation and degradation pressure on existing old-growth forests [8]. Given the importance of secondary forests, a detailed understanding of the successional processes governing the development of their structure, soil properties and species composition is critical. However, despite numerous studies on successional processes, substantial uncertainty persists regarding their growth rates [5], nutrient dynamics [9], and the interactions between land use history and successional trajectories [8]. This is, in part, a result of the numerous factors influencing regeneration, including soil type [3] and nutrient availability [10], previous land use intensity [11], fire history [12], topography [13], and distance to seed trees [14].

During secondary forest succession, changes in stand structural attributes occur in sync with changes in soil properties and species composition. Succession is typically divided into distinct structural phases characterized by a unique suite of species [15] traditionally divided into successional/functional guilds [16]–[19]. The first phase of succession lasts only 1–5 years and is dominated by herbs, shrubs and climbers. During the second phase, from approximately 3–30 years following abandonment, pioneer species, with low wood density, rapid growth rates and high light requirements, develop a short stature closed canopy resulting in phase one species being shaded out. A transition then occurs within the species regeneration guild from short-lived ‘pioneers of initiation’ to longer-lived ‘pioneers of exclusion’ [20], which while still having high light requirements and rapid growth rates, are able to gain taller statures more typical of a mature forest. In the final phase slow growing shade tolerant species with high wood density replace the pioneers, as most pioneer species seedlings are incapable of growing in the increasingly shaded understory [21], [22], resulting in a composition approaching that of mature forest [17], [23]. Waring and Running (2007) [24], and see Oliver and Larson (1990) [25] as applied to tropical successional systems in Chazdon (2008) [26], refer to the structural phases of forest succession as stand initiation, stem exclusion and understory reinitiation phases, with the final old growth stage, in both stand structure and composition, being reached 100 to 400 years post-abandonment.

At the species scale, forest succession theory has typically grouped species according to regeneration requirements into species successional categories or guilds [27]. Although common, this approach is limited and studies are beginning to investigate the dynamics and ecological implications of approaches incorporating more detailed species successional classifications [28], [29]. Such studies have the potential to elucidate gradients of change in successional species not easily seen when using categorical classifications [23]. New approaches have been undertaken by increasing the number of successional categories [28], referred to as plant functional types, and by developing continuous gradients of successional status using multi-variate methods [23], [30]. An improved understanding of how successional status affects leaf traits in secondary forests is necessary given: (a) an increased interest in linkages between plant functional traits and species assembly processes [31], [32]; and (b) the increasing use of leaf traits as indicators of ecosystem nutrient cycling and limitation [33].

Foliar properties, including nutrients and isotopes, are being increasingly used to describe the dynamics of plant communities [34], [35] and may provide new insights into community successional dynamics [31]. Within most terrestrial ecosystems, nitrogen (N) and phosphorus (P) availability are the primary limiters of plant growth [34] and the foliar N:P ratio has been of particular focus [36]. Its use has highlighted changes from a conservative N cycle in early secondary sites to a conservative P cycle later in succession [37], with an increase in the foliar N:P ratio being used to indicate a shift to P limitation of ecosystem processes [38]. Co-limitation by N and P is also possible [39], similar to that which can occur during primary succession [40]. Further insights into ecosystem dynamics have been revealed by combining foliar nutrient concentrations with carbon and nitrogen stable isotopes. The carbon isotope ratio (δ^13^C) is representative of leaf intercellular processes and water use efficiency, integrating photosynthetic activity throughout the leaf’s lifespan (41). Foliar δ^13^C is correlated with a broad range of plant functional characteristics, including leaf size and thickness, stomatal density, and gas exchange metabolism [41] and leaf mass per area (LMA; [42]). The nitrogen stable isotope ratio functions as more of an ecosystem scale integrator determined by internal processes and varying input-output balances, with decreasing foliar δ^15^N generally representing a tightening or closure of the N cycle [43].

Although leaf traits have potential to improve our understanding of forest succession dynamics, few studies have been conducted on the factors, including stand or soil properties, constraining leaf trait variation [28], [41], [43]. In particular, few studies have investigated the drivers - including soil isotope variation [44], [45] - of foliar isotope variation within different successional tree species during succession [46], [47]. We use a high-resolution forest succession chronosequence following slash-burn agriculture to evaluate the biotic and abiotic predictors of leaf trait variation in 20 tropical tree species encompassing a continuous gradient from early to late successional status. Biotic predictors include stand structural characteristics, taxonomic and phylogenetic analyses, and species successional position, calculated as the stand age at which each species becomes most abundant. Abiotic predictors include a suite of soil properties, including fertility and structure measurements. Our overarching interest is whether leaf trait variation during forest succession is explained principally by changes in: (a) stand age, (b) forest structure, or (c) soil properties, or, alternatively, by (d) shifts along a continuous gradient of species varying in life strategy. In addition, we consider phylogenetic signal as a predictor of leaf trait variation. Results from this study are relevant to better understanding forest regeneration following disturbance, a carbon sink of global importance, as well as forest community and species dynamics in general.

## Materials and Methods

### Study Sites

This study was carried out in the community of Molienda (municipality of Bolpebra and Department of Pando) in the Bolivian Amazon (11°26′28.189″ S, 69°09′30.06″ W), with the assistance of community members who worked with us, allowed us access to their properties, and shared their extensive knowledge of the area. The forest is considered lowland tropical moist forest with mildly undulating topography, has a mean annual rainfall of 1800 mm, and has a pronounced dry season extending from May to September [48]. In this community, most households rely on slash-and-burn agriculture as their principal food source, with preference to opening agricultural areas within primary forest. Patches of current slash-and-burn agriculture, usually less than 3 ha in extent (hereafter referred to as agriculture), and successional forests growing on abandoned agricultural fields are dispersed throughout the primary forest, which dominates the landscape. Forest stands used in this study were identified through interviews with long-term residents. Information of each stand was cross-validated using important historical events and through triangulation via interviews with multiple community members. Only stands with similar topography, hydrology, and land use history were included. Stands were distributed widely throughout the landscape (>500 m apart) to minimize spatial auto-correlation. In total, 15 successional stands <3 ha in extent and surrounded by primary forest, with ages ranging from 4–47 years, and two primary forest stands were identified, giving this chronosequence among the highest temporal resolutions and range identified in our literature review. All stands were initially primary forest, which were cleared, burned and then used for growing beans, corn, rice and yucca for 2–3 years prior to abandonment. No stands underwent wildfires, logging, or had been reentered for agricultural use post-abandonment. These stands, being first-cycle, are therefore representative of the lowest intensity of proceeding land use in the Amazon [11].

### Forest Structure and Composition

Forest inventories were conducted using one 10×80 m belt transect randomly selected within each stand with the requirement that all transect area was at least 20 m from the stand edge. All trees, both living and dead, >2 m in height were measured. Botanical samples were collected and brought to the Centro de Investigación y Preservación de la Amazonia (CIPA) herbarium in Cobija, Bolivia for identification. All trees were mapped to Cartesian coordinates within each transect and, for each tree, we quantified diameter at breast height (DBH; 1.3 m; cm), height (m), and crown exposure (CE), defined using a five-point scale (Clark and Clark 1992) in which 1 = no direct light or low amount of lateral light, 2 = intermediate or high amount of lateral light, 3 = vertical light in part of the crown, 4 = vertical light in the whole crown, and 5 = exposed crown with direct light coming from all directions (i.e., emergent). Liana infestation was defined for each tree using a scale of 1 = none, to 4 = completely covered. The approximate percentage of each tree’s crown volume was estimated for the following four categories: new, senescent, and mature leaves or no leaves present. Wood density was estimated for all tree species at the highest taxonomic resolution possible using the web-based wood density database [49], and information derived from Nogueira et al. (2007) [50] and Fearnside (1997) [51]. In cases where species identification was not available (29% of living stems), individuals were given the mean calculated wood density of the forest stand. Biomass (kg) was calculated using the equation for tree biomass described in Chave et al. (2005) [52]:
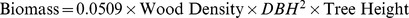
For each stand we also calculated the average and maximum tree height and DBH which we refer to as height_avg_, height_max_, DBH_avg_, and DBH_max_, respectively, and used the mean value of all trees within each stand to describe the other stand structural variables.

**Figure 1:**
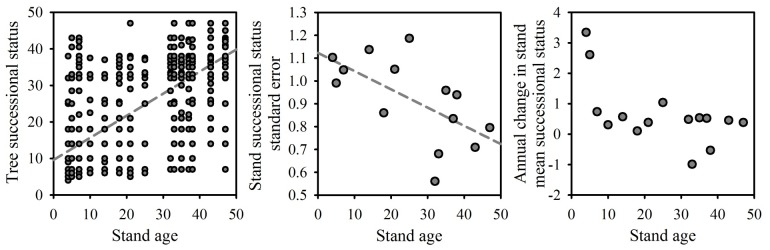
Linear relationship between stand age (years) and species successional status mean (years; left), standard error (SE; middle) and annual change (years; right). Stand values are calculated using all tree stems within each secondary forest stand.

We used the Shannon Weiner index to compare compositional diversity among forest stands. The Chao-Jaccard (CJ) dissimilarity index (zero = no dissimilarity, one = complete dissimilarity) was run between all stand ages (excluding dead stems) [53] using the *vegdist* and *mantel* tests in the ‘vegan’ package in R. Significance of compositional differences among all stand ages was tested using the Pearson method of the Mantel test with 1000 permutations. We developed a continuous metric, termed species successional status, calculated separately for each tree species as the median stand age in which the each species occurred weighted according to the number of stems of each species occurring within each study plot. Species occurrences were also used by Chazdon et al. (2011) [54] to classify generalist and specialist tree species in tropical habitats. Species successional status had a significant positive relationship with stand age (Adj-*R* ^2^ = 0.48, *P*<0.0001, *N* = 1479; Figure 1), while the lower *R* ^2^ value indicated that tree species occurred across a wide range of stand ages enabling the subsequent comparative analyses of the effects of stand age and species successional status on leaf trait variation. This approach differs from that of Peña-Claros (2003) [23], and used by Poorter (2004) [30], which used correspondence analysis to assign a value of 0 (earliest) to 100 (latest) for successional status, as it directly provides a successional status age for each species enabling direct comparison with measurements of stand structure and soil properties. Species data from primary forest stands were not used in the calculation of successional age as stand age was unknown.

### Soil Properties

Soil cores (2×4″; AMS, Inc., American Falls, ID) were collected for three randomly chosen locations at depths of 0–10, 10–20, and 20–30 cm. Soil samples were then aggregated for each depth in the field, oven dried at 50°C for 72 hours, lightly ground and sieved to 2 mm to remove coarse particles, including roots and stones. Mineral fractions weighing ∼150 grams were placed into 50 ml polypropylene centrifuge tubes for transportation to the Department of Global Ecology for further analysis. pH was measured on fresh soil samples in solution immediately following collection using a hand-held pH meter (Hanna Instruments, Inc.,Woonsocket, RI). Bulk density was determined using the approach described in Elmore and Asner (2006) [180] using the soil corer to obtain a soil from a known volume for each depth. Bulk density samples were oven dried at 70°C for 96 hours, and sieved to 2 mm to remove roots and stones. The mineral fraction of the soil sample was weighed using a portable electronic scale (Ohaus, Inc., Pine Brook, NJ). The volume of fractions larger than 2 mm was recorded using the displacement method. The bulk density per depth was calculated as the mineral fraction sample mass divided by the volume (170–206 ml), adjusted for the volume of the large fraction (mean±standard deviation was 2.6±1.4% of volume). Soil mass was calculated as Mg ha^−1^ to 30 cm depth using the average of the three bulk density measurements and adjusted for the mass >2 mm.

The soil samples were ground to a fine powder using a Wiley Mill and elemental content (%C; %N) for carbon and nitrogen and δ^13^C and δ^15^N isotope ratios were quantified using a Carlo Erba EA 1110 C:N combustion (NC2500, CE Instruments, Milan, Italy) coupled with an isotope ratio mass spectrometer (IRMS Delta Plus, Finnigan Mat, San Jose, CA) operating in a continuous flow mode. Standards used for carbon and nitrogen isotopes are PDB and AIR, respectively. Data are expressed in δ (‰) notation [177], [178] where:
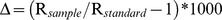
with R equal to the ratio of ^13^C:^12^C or ^15^N:^14^N for the sample and standard. In this context, a positive δ value means the sample has more of the heavier isotope than the standard and vice-versa. We use the terminology of enrichment (i.e., positive value or less negative trend; becoming heavier) or depletion (i.e., negative value or more negative trend; becoming lighter) of the heavier isotope versus the standard as described by Dawson et al. (2002) [41]. Individual soil depths were then aggregated and homogenized to get average 0–30 cm depth soil samples. These samples were measured for extractable phosphorus using the weak Bray and Sodium Bicarbonate methods (ppm; P1 and P2, respectively), soil pH (saturated paste method), extractable cations (ppm; K, Mg, Ca, Na and H) using 1.0 ammonium acetate @ pH 7.0, and soil texture using NaHexametaphosphate+hydrometer (%) at A&L laboratories (Modesto, CA). Cation exchange capacity (CEC) was calculated as the sum of K, MG, Ca, Na and H (meq/100 g). All variables were converted from meq/100 g, % or ppm to a 0–30 cm per hectare scale using the soil mass (kg ha^−1^).

### Foliar Properties

Tree species selected for foliar analyses were identified to encompass a wide taxonomic range and to be present in as many age stands as possible. Two to three top of canopy trees of each study species were randomly chosen from each stand provided the particular species was present. In total, 149 tree individuals encompassing 20 species, or approximately 10% of all tree species, were selected. From these individuals we collected 10–15 fully expanded mature leaves from two separate locations within the full sunlight portion of each individual’s crown either by hand or using a shotgun. Leaf samples were oven dried at 60°C for 72 h, sealed in plastic bags and stored in an air-conditioned room prior to transportation to the Carnegie Institution’s Department of Global Ecology at Stanford University. Foliar samples were aggregated to the scale of sample tree and ground to a fine powder using a Wiley Mill (Thomas Scientific, Swedesboro, NJ). Foliar N (TKN; mg g^−1^) and P (TKP; mg g^−1^) were extracted using a sulfuric acid/hydrogen peroxide digest and quantified using simultaneous colorimetric N and P analyses on a rapid flow autoanalyzer (OI Analytical, College Station, TX), using the ammonium molybdate ascorbic acid method [179]. Elemental content (%C; %N) for carbon and nitrogen and δ^13^C and δ^15^N isotope ratios were quantified on leaf samples aggregated from each tree using a C:N combustion analyzer coupled with an isotope ratio mass spectrometer as described in the soil methods. Foliar C:N values were calculated using % values and N:P ratios were calculated using Alpkem values.

### Statistical Analysis

Statistical analyses for this and all following sections were carried out using JMP v.7.0.1 (SAS Institute, Inc.) and in R v.2.9.2 (http://www.R-project.org). The raw stand, tree, soil and foliar data used in this study is provided as File S1. Summary statistics in the following sections refer to mean ± standard deviation. We use an alpha-level for significance testing of 0.05 throughout, but provide data on less significant findings for general interpretation. First, we measured overall changes in stand structural and soil properties through regressions versus stand age. Soil analyses are aggregated soil depth samples, with the exception - when indicated - of a multiple regression analysis for soil δ^13^C and δ^15^N including stand age, soil depth (5, 15 and 25 cm) and their interaction as predictor variables. While we compared secondary forest values with those from the primary forest plots using a two-sided t-test, results from these analyses were used for descriptive purposes only given the small primary forest sample size (*N = *2 for soils and stand values). Second, we investigated relationships between leaf traits and a suite of predictor variables. Leaf traits were treated as response variables and included: (a) %C, %N, and their ratio; (b) N (mg g^−1^), P (mg g^−1^), and their ratio; and (c) the stable isotope ratios δ^13^C and δ^15^N. Potential predictors of leaf trait variation were: (a) stand age, (b) stand structure, (c) soil properties, and (d) species successional status. Third, to test for changes in plant isotope fractionation during succession – potentially indicative of changes in the importance of mycorrhizal fungi [43] – linear regressions were run between foliar-minus-soil isotope values, referred to as Δδ^13^C_plant-soil_ or Δδ^15^N_plant-soil_ [55], and stand age and species successional status.

Whereas stand age and species successional status were individual values, the stand structure and soil properties groups were each composed of 18 unique, but often correlated, variables. To summarize each of these groups we used the first two axes of separate Principal Components Analyses (PCAs) [56]. To identify the most important predictor variables for each leaf trait we used the *bestglm* command (best subsets approach) based on the Akaike Information Criteria (AIC) in R and then used changes in the adjusted *R* ^2^ value to select the best number and combination of predictor variables for each leaf trait. Pearson correlations were used to assess relationships among all predictor variables. We further explored relationships between leaf traits, individual stand, and soil variables through regression analysis. To directly test the importance of stand age versus species successional status on leaf trait variation we used the ANOVA command in R to compare linear regression models including only stand age or species successional status to a model including both variables. To test if individual species followed the same relationships as those found across the species community we used linear regressions between leaf traits and stand age for four species (*Cecropia polystachya*, *Miconia* sp., *Jacaranda cuspidifolia* and *Inga* sp.) representing early to later successional statuses and occurring across a wide range of stand ages. In the case of foliar δ^13^C, in which intra-species patterns opposed those at the community scale, we ran an additional multiple linear regression model using stand age, species successional status and their interaction as predictor variables. Data for leaf trait analyses were transformed, when significantly different from normal as indicated using the *shapiro.test* command in R, using either the Box Cox, logarithmic, square root, or exponential transformation in R.

**Figure 2:**
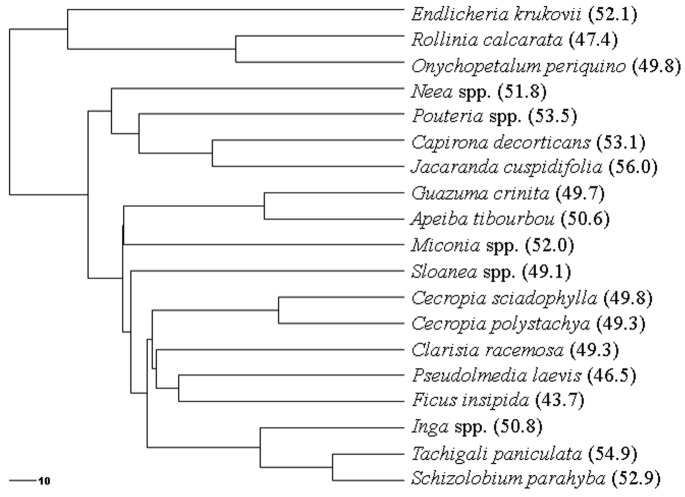
Ultrametric tree of species used for leaf trait phylogenetic signal analyses. Foliar %C is provided after the species name as it was the only trait identified as having significant phylogenetic signal. The scale bar (lower left corner) represents 10 million years.

Third, we tested separately for taxonomic and phylogenetic sources of leaf trait variation, similar to the approach used by Swenson and Enquist (2007) [57]. Taxonomic differences in each leaf trait were tested using a Kruskal-Wallis test in R. A phylogram of phylogenetic relationships among our study species, based on molecular data compiled into a mega-tree, was constructed using Phylomatic (www.phylodiversity.net/phylomatic/), which is a standard approach used in over 46 peer-reviewed articles (www.citeulike.org/group/4921/library/). Further relationships among the Fabaceae were resolved following the Tree of Life Web Project (www.tolweb.org/Fabaceae) and Wojciechowski et al. (2004) [58], and among Moraceae following Zerega et al. (2005) [59]. Angiosperm node ages were calculated from Wikstrom et al. (2001) [60] and Hedges et al. (2006) [61] (www.timetree.org). The resultant phylogeny had five (of 18) soft polytomies the ages of which were estimated using Phylocom BLADJ (Branch Length ADJuster; www.phylodiversity.net/bladj; Webb et al. 2008 [62]), following which the phylogeny was converted to an ultrametric tree (i.e., branch lengths consistent with estimated relative time of divergence; Figure 2).

Tests for phylogenetic signal (i.e., do related taxa have more similar leaf traits) were run separately for each leaf trait and for species successional status using the *phylosignal* module of the *picante* package in R [63]. The *phylosignal* module tests for the presence of phylogenetic signal by comparing observed patterns of a leaf trait to a null model produced by randomly shuffling taxa labels across the tips of the phylogenetic tree [64], providing a *P*-value of signal significance, which does not provide information about trait evolution, and a *K*-statistic which tests for evolutionary processes by comparing trait data to an evolutionarily null model in which a *K*-statistic of one is equal to a Brownian motion model of evolution [64]. Although *K*-statistic values greater than one indicate conservatism of traits versus random or convergent evolution (∼0), we do not attempt to make any evolutionary arguments in this study [65], given the incomplete status of our phylogenetic tree [66].

## Results

### Forest Structure and Composition

**Figure 3:**
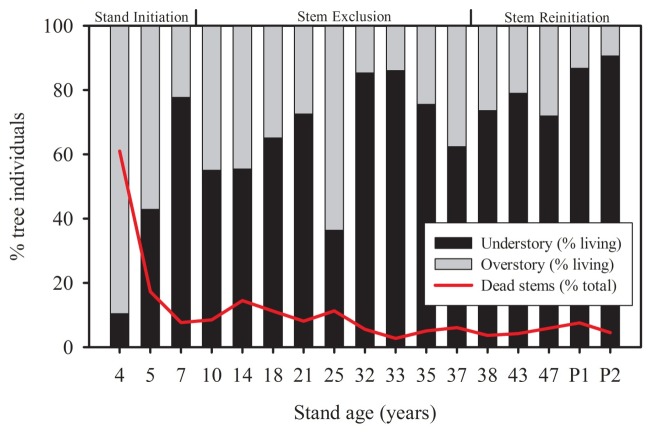
Percentage total living trees in understory and overstory crown exposure (CE) positions; 1–3, and 4–5, respectively. Stand development phases (top) correspond to those described by Waring and Running (2007) [24] and Oliver and Larson (1990) [25].

**Figure 4:**
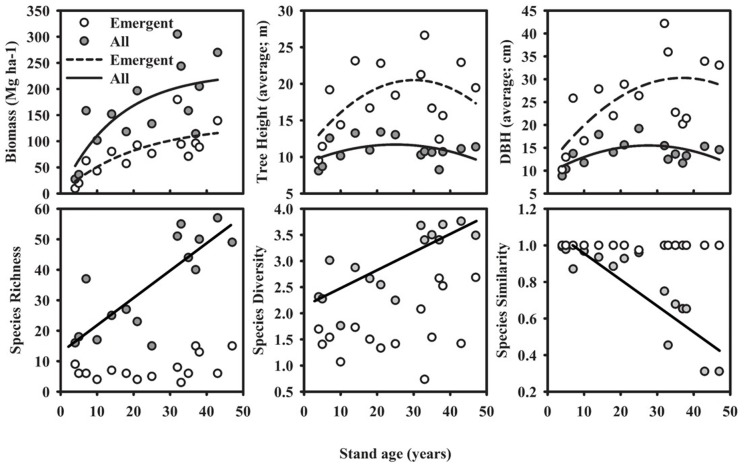
Stand structural and species characteristics for successional forest study sites. Species diversity is calculated using the Shannon-Weiner metric and species similarity is calculated using a Chao-Jaccard index versus primary forest composition. Michaelis-Menten relationship is shown between biomass and stand age, while quadratic are shown between tree height and diameter at breast height (DBH) and stand age.

Forest structure and species richness and diversity information for all study sites are summarized in Table 1. Data were collected from 17 stands encompassing secondary forest ages 4 to 47 and two primary forest stands. We identified 205 species from 1,892 individual trees. The most abundant species was *Jacaranda cuspidifolia* (Bignoniaceae) which had 157 individuals and dominated the developing stand stage. One hundred and thirty eight of our species were represented by less than five individuals, while 78 were represented by only one. The average tree crown contained 65%, 8% and 4% of mature, senescent and new leaves, respectively, while 23% of the crown volume remained vacant. Three distinct phases were identified within our chronosequence (Figure 3), corresponding to stand initiation, stem exclusion and understory reinitiation stages [24], [25]. During phase one the percentage of dead trees decreased rapidly from greater than 60% to less than 10% in the 4 and 7 year old stands, respectively. Phase two demonstrated a stem exclusion peak as shown through an increase, then decrease, in understory stem density. Phase three showed a steady increase in understory stem density with little stem mortality. Biomass increased according to a positive Michaelis-Menten asymptotic relationship with stand age during forest succession, while both tree height and DBH for the entire community and for emergent trees only, exhibited significant linear and quadratic relationships (Figure 4). Peaks in tree height and DBH were found between 30–40 years post-abandonment. The Pearson mantel statistic showed significant changes in community composition among the study stands for the entire community (*R = *0.37, *P = *0.001, *N* = 1892) and within emergent trees only (*R = *0.43, *P = *0.001, N = 1892). While the entire tree species community – dominated by the understory - became more similar to primary forest species composition (*R^2^* = 0.75, *P*<0.0001, *N = *15) during succession, no such pattern was shown in the emergent trees, which remained completely dissimilar (i.e., Chao-Jaccard = 1) at all stand ages. Stand age had significant positive correlations with both tree community species richness (Adj-*R^2^* = 0.62, *P*<0.0001, *N = *15) and species diversity (Adj-*R^2^* = 0.64, *P*<0.0001, *N = *15), with positive trends between stand age and emergent tree species richness (Adj-R^2^ = 0.15, *P* = 0.0819, *N* = 15) and diversity (Adj-R^2^ = 0.15, *P* = 0.0824, *N* = 15). Community species richness was also significantly positively correlated with community species diversity (Adj-*R* ^2^ = 0.93, *P*<0.0001, *N* = 14). The standard error of species successional statuses, calculated across all stems in each stand, had a significant negative relationship with stand age (*R^2^* = 0.38, *P*<0.0001, *N = *14; Figure 1). While rate of taxonomic compositional change was greatest following 20 years, as indicated through species similarity to primary forest (Figure 4), the rate of change in stand mean successional status was greatest prior to 10 years after which it reduced greatly (Figure 1).

**Table 1:** Stand characteristics of all successional and primary (Pr) forest study sites (mean±std. dev.).

	Stand age (years)																
	4	5	7	10	14	18	21	25	32	33	35	37	38	43	47	Pr-1	Pr-2
Tree density (# ha^−1^)	1537.5	1375.0	1637.5	1912.5	950.0	1450.0	1237.5	775.0	1350.0	1837.5	1237.5	1025.0	1375.0	1487.5	1700.0	1325.0	1387.5
Dead trees (# ha^−1^)	937.5	237.5	125.0	137.5	137.5	162.5	100.0	87.5	75.0	50.0	75.0	62.5	50.0	62.5	100.0	100.0	62.5
Basal area (m^2^ ha^−1^)	10.6	13.2	32.1	24.8	33.4	32.1	31.6	28.2	43.7	36.1	27.3	17.3	30.5	43.3	54.0	34.5	36.3
Biomass (Mg ha^−1^)	27.3	36.1	158.3	101.8	152.0	118.2	196.6	133.4	304.9	243.7	158.4	114.3	204.9	269.8	460.5	282.3	325.6
CE (avg±std. dev.; 1–5)*	4.5±0.7	3.5±1.5	2.3±1.3	3.2±1.4	3.1±1.5	2.9±1.4	2.6±1.4	3.6±1.4	2.1±1.3	1.9±1.2	2.6±1.3	2.8±1.6	2.7±1.3	2.5±1.3	2.6±1.4	1.9±1.2	1.7±1.0
Tree height (avg±std. dev.; m)	8.1±2.3	8.7±3.0	12.6±4.9	10.2±3.9	13.3±7.2	10.9±5.7	13.4±6.9	13.0±6.0	10.3±5.0	10.7±6.7	10.6±6.6	8.2±4.3	10.7±6.9	11.1±5.9	11.4±6.5	11.8±6.1	11.1±6.3
Tree height (max; m)	14	16	25	19	28	27	30	26	28	30	27	30	28	27	38	30	36
DBH (avg±std. dev.; cm)**	8.9±3.0	10.3±4.0	13.7±7.9	11.7±5.3	17.9±11.4	14.0±9.3	15.6±9.1	19.2±9.8	15.5±13.2	12.5±9.8	13.6±9.8	11.6±9.0	13.2±10.4	15.3±11.8	14.6±13.9	13.5±12.3	13.3±12.6
DBH (max; cm)**	17.7	20.0	59.3	30.0	69.0	67.0	45.8	52.0	78.5	49.5	55.2	73.0	50.8	52.8	102.0	82.0	78.3
Lianas (avg±std. dev.; 1–4)	2.1±1.0	1.5±1.1	1.3±0.8	1.6±1.0	1.8±1.0	1.8±1.2	1.7±1.1	1.5±1.0	1.5±0.9	1.7±1.1	1.8±1.1	2.5±1.3	1.7±1.0	1.3±0.7	1.8±1.1	1.4±0.6	1.5±0.9
Mature leaves (avg±std. dev.; %)	65.0±23.3	56.2±23.5	66.0±22.3	67.0±25.5	52.7±31.1	65.2±31.9	55.1±31.1	41.1±32.7	72.6±25.5	75.0±23.3	65.8±31.4	75.6±22.7	72.0±25.5	62.4±28.6	76.3±27.8	70.2±23.4	72.8±22.0
New leaves (avg±std. dev.; %)	5.4±9.7	4.1±8.7	3.4±9.0	3.7±12.6	4.7±18.3	5.7±14.3	5.7±12.0	4.4±13.4	5.0±17.7	3.4±11.4	6.6±17.7	0.9±5.7	2.4±7.0	2.4±11.6	2.7±11.1	6.7±13.8	4.4±10.9
Senescent leaves (avg±std. dev.; %)	13.5±10.8	14.0±12.1	7.0±8.6	10.3±12.9	10.5±11.5	6.2±9.1	7.6±9.6	8.6±9.1	6.5±10.0	6.7±9.8	6.6±9.0	6.2±10.3	7.5±9.1	9.7±10.1	5.1±8.8	6.0±12.7	4.5±7.7
No leaves (avg±std. dev.; %)	16.0±19.4	26.0±22.8	23.6±20.4	19.0±21.6	32.2±30.9	22.8±27.1	31.7±29.6	46.0±32.1	15.9±17.7	15.0±18.7	21.1±26.6	18.1±22.6	18.2±22.1	25.6±27.2	16.0±21.6	17.0±15.2	18.2±16.7
Wood density (avg±std. dev.; kg m^−3^)	456±112.3	402.7±130.4	496.3±124.2	521.3±95.2	459.2±128.3	464.8±132.9	485.6±107.1	466.9±97.9	611.9±91.3	654.0±177.3	526.1±91.0	620.8±96.9	578.1±88.3	579.2±91.1	601.2±83.5	601.3±59.2	606.4±83.1
Richness (# sp transect-1)	16	18	37	17	25	27	23	15	51	55	44	40	50	57	49	38	43
Diversity (Shannon-Weiner)	2.3	2.3	3.0	1.8	2.9	2.7	2.6	2.3	3.7	3.4	3.5	3.4	3.7	3.8	3.5	3.3	3.5
CJ vs. primary***	0.99	0.98	0.87	0.97	0.94	0.89	0.93	0.96	0.75	0.45	0.68	0.65	0.65	0.31	0.31		

*Crown Exposure (CE) index, **Diameter at Breast Height (1.3 m), ***Chao-Jaccard community composition similarity versus primary forest sites.

Stand PCA axes 1–3 explained 47%, 24% and 9% of variation across all stand structural variables, respectively (Table 2 stand axes). Stand PCA axis one had strong positive correlations with biomass, basal area, species diversity and height_max_ and negative correlations with number of dead trees and the % crown with senescent leaves. As the strongest correlations were with biomass, we refer to this as the biomass axis. PCA axis two had strong positive correlations with % crown with no leaves, DBH_avg_ and height_avg_ and negative correlations with tree density, liana infestation, and % crown with mature leaves. As the strongest correlations were with height_avg_ and DBH_avg,_ we refer to this axis as the structure axis. While the biomass axis had a significant positive linear relationship with stand age (PCA1 = −4.20 + 0.17 * Stand age, *R* ^2^ = 0.71, *P*<0.0001, *N* = 15) the structure axis did not. Secondary forests, as compared to primary forests, had significantly lower biomass (178±110 vs. 304±31), wood density (528±75 vs. 604±4), DBH_max_ (54.8±22.1 vs. 80.2±2.6), % crown in mature leaves (64.5±9.8 vs. 71.5±1.84) and greater mean crown exposure class (2.86±0.65 vs. 1.8±0.14), liana infestation (1.71±0.30 vs. 1.45±0.07) and % crown in senescent leaves (8.4±2.7 vs. 5.3±1.1).

**Table 2:** Eigenvectors of stand and soil Principal Components Analysis (PCA) axes.

Stand biomass axis			Stand structure axis			Soil texture axis			Soil fertility axis		
Stand properties		PCA 1	Stand properties		PCA 2	Soil properties		PCA 1	Soil properties		PCA 2
Richness	+	0.30711	Mean DBH	+	0.43933	Clay	−	0.37631	Nitrogen	+	0.44133
Biomass	+	0.30329	No leaves	+	0.42061	Sand	+	0.33806	K	+	0.37087
Max height	+	0.2997	Mean height	+	0.4199	C	+	0.33283	Carbon	+	0.35208
CE	−	0.29305	Mature leaves	−	0.36626	P1	+	0.31279	H	+	0.32176
Diversity	+	0.29001	Tree density	−	0.2818	Silt	−	0.30596	δ^15^N	−	0.31279
Chao-Jaccard	−	0.28963	Liana	−	0.21519	CEC	+	0.2773	Mg	+	0.28983
Wood density	+	0.28943	Dead trees	−	0.2048	P2	+	0.2607	CEC	+	0.2672
Basal area	+	0.28547	Wood density	−	0.17084	Base Saturation	+	0.25635	δ^13^C	+	0.25618
Senescent leaves	−	0.28039	Basal area	+	0.15914	Mg	+	0.23177	Na	+	0.1867
Max DBH	+	0.27384	New leaves	+	0.15187	δ^13^C	−	0.21944	Sand	−	0.18151
Dead trees	−	0.2258	Max height	+	0.14433	δ^15^N	−	0.20182	Clay	+	0.17175
Mature leaves	+	0.20452	Max DBH	+	0.11745	Na	−	0.17779	Silt	+	0.12216
New leaves	−	0.14245	Richness	−	0.11551	Soil mass	−	0.14391	P2	−	0.04887
No leaves	−	0.12287	Chao-Jaccard	+	0.11432	Bulk density	−	0.14209	P1	+	0.04834
Mean DBH	+	0.10854	Senescent leaves	−	0.06739	Carbon	+	0.0838	Soil mass	−	0.03816
Mean height	+	0.07784	Biomass	+	0.06094	H	+	0.06165	Bulk density	+	0.03518
Tree density	+	0.0664	Diversity	−	0.05918	K	−	0.05983	Base Saturation	−	0.03317
Liana	−	0.02621	CE	−	0.03973	Nitrogen	+	0.04247	C	+	0.02439

Stand and soil properties and units are provided in Tables 1 and 3, respectively.

### Soil Properties

**Figure 5:**
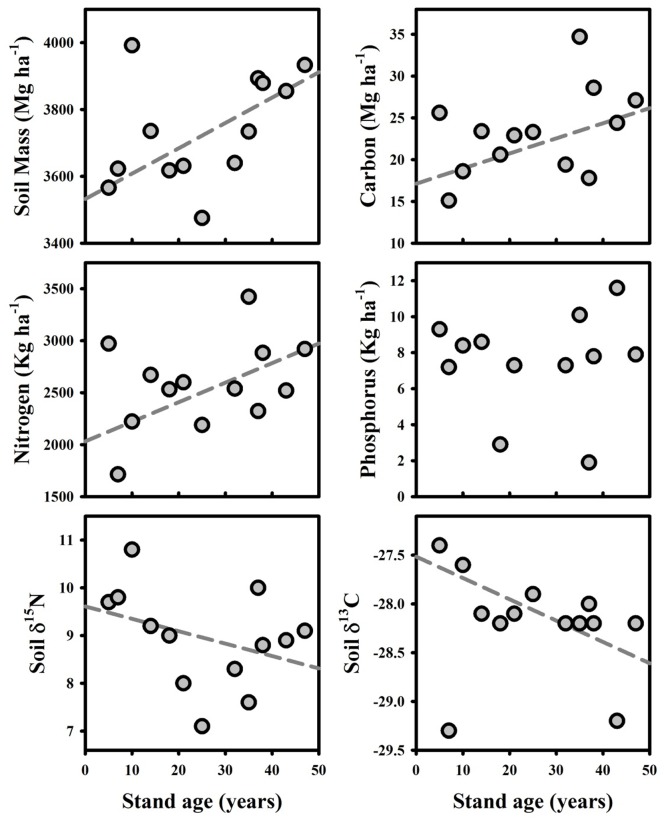
Selected soil properties for successional forest study sites. Significant linear regressions are shown in grey (*N* = 13). Linear regressions for soil δ^15^N were significant using separate depth points (*N = *39) and for soil δ^13^C following exclusion of the 7 year old stand outlier (*N* = 12).

Soil properties for all study sites are summarized in Table 3 and Figure 5. Significant relationships were found between stand age and soil δ^13^C (negative, *R* ^2^ = 0.45, *P = *0.0171, *N = *12), CEC (positive, *R* ^2^ = 0.31, *P = *0.0592, *N = *12) and soil N (positive, *R* ^2^ = 0.34, *P = *0.0461, *N = *12). While significant positive relationships were found between stand age and total 0–30 cm depth soil mass (Mg ha^−1^) (*R* ^2^ = 0.55, *P = *0.0058, *N = *12), and individually for 10–20 cm (*R* ^2^ = 0.55, *P = *0.0058, *N = *12) and 20–30 cm (*R* ^2^ = 0.55, *P = *0.0058, *N = *12), no significant relationship was found for 0–10 cm. The percentage of soil mass >2 mm (mean±STD of 2.47±0.74%) did not show significant changes with stand age, although a trend of an increasing fraction in the 0–10 cm depth and decreasing in the 20–30 cm depth was seen.

**Table 3:** Soil properties for all successional and primary (Pr) forest study sites.

	Stand age (years)														
	5	7	10	14	18	21	25	32	35	37	38	43	47	Pr-1	Pr-2
Carbon (C; Mg ha^−1^)	25.6	15.1	18.6	23.4	20.6	22.9	23.3	19.4	34.7	17.8	28.6	24.4	27.1	25.7	20.4
Nitrogen (N; kg ha^−1^)	2971.6	1714.9	2222.3	2670.9	2532.5	2600.1	2189.5	2538.8	3422.9	2322.9	2883.6	2521.3	2920.4	3210.8	2365.5
δ^15^N	9.7	9.8	10.8	9.2	9.0	8.0	7.1	8.3	7.6	10.0	8.8	8.9	9.1	9.2	9.1
δ^13^C	−27.4	−29.3	−27.6	−28.1	−28.2	−28.1	−27.9	−28.2	−28.2	−28.0	−28.2	−29.2	−28.2	−27.1	−27.4
Phosphorus (P1; kg ha^−1^)*	9.3	7.2	8.4	8.6	2.9	7.3		7.3	10.1	1.9	7.8	11.6	7.9	9.9	2.8
Phosphorus (P2; kg ha^−1^)**	10.7	10.9	12.0	11.2	10.9	10.9		7.3	11.2	3.9	7.8	15.4	15.7	7.9	7.0
Calcium (Ca; kg ha^−1^)	253.0	138.7	256.4	305.4	279.5	348.9		690.0	418.7	180.6	223.1	1465.1	253.4	269.4	1366.4
Potassium (K; kg ha^−1^)	284.6	93.7	87.6	148.3	128.4	235.2		194.9	196.2	213.1	108.5	98.2	209.0	264.8	141.6
Magnesium (Mg; kg ha^−1^)	201.9	53.2	82.5	171.4	152.4	150.8		254.7	169.4	121.4	80.9	196.0	107.9	134.3	357.2
Sodium (Na; kg ha^−1^)	27.8	23.2	34.3	34.7	31.1	53.4		29.5	31.7	44.4	35.7	28.1	42.9	45.8	41.2
Hydrogen (H; kg ha^−1^)	32.3	21.9	32.2	26.4	36.5	40.3		73.4	48.9	31.4	31.3	27.2	67.4	47.8	17.6
CEC (kmol ha^−1^)***	69.8	36.4	55.3	60.8	67.3	78.1		134.5	89.8	57.5	53.2	120.0	95.6	80.6	120.5
Base saturation (%)	54.0	40.3	42.2	57.0	46.2	48.9		45.9	45.9	45.9	41.6	77.5	30.1	41.2	85.5
Sand (Mg ha^−1^)	1797.2	2478.1	2331.4	2256.3	1823.4	2338.6	1682.1	2344.1	1956.7	1261.4	1800.0	2559.8	2218.4	1358.0	2251.3
Silt (Mg ha^−1^)	1155.4	666.6	894.2	836.8	1099.8	668.2	1195.5	669.7	1060.5	1572.8	1334.5	863.6	881.0	1515.9	713.1
Clay (Mg ha^−1^)	613.3	478.2	766.5	642.5	694.6	624.6	597.8	626.1	716.9	1058.9	744.8	431.8	833.8	1073.8	531.4
Soil mass (Mg ha^−1^)€	3565.9	3623.0	3992.2	3735.6	3617.8	3631.4	3475.4	3639.9	3734.1	3893.1	3879.3	3855.1	3933.2	3947.8	3495.8
Bulk density (Avg. 0–30 cm; g cm^−3^)	1.233	1.235	1.357	1.300	1.235	1.237	1.180	1.269	1.303	1.336	1.323	1.309	1.338	1.399	1.183

*Weak Bray Extraction,

**Strong Bray Extraction,

€ Adjusted for mass >2 mm,

***Cation Exchange Capacity calculated as the sum of Ca, K, Mg, Na and H (meq/100 g).

Strong positive trends were found, significant following exclusion of the youngest successional stand due to extensive charcoal from the recent burn, between stand age and soil carbon (Mg ha^−1^), (*R* ^2^ = 0.31, *P = *0.0589, *N = *12) - but not soil organic matter (%) or %C. A negative trend was identified between soil base saturation and stand age (*R* ^2^ = 0.29, *P = *0.0854, *N = *11). No significant relationships were found between stand age and soil phosphorus [as measured through P1 and P2 methods], or sand, silt and clay content (Mg ha^−1^), although soil P1 had a significantly negative relationship (with a trend in P2) with soil clay content (*R* ^2^ = 0.38, *P = *0.0321, *N = *12) which was not correlated with stand age. While soil %C had no correlation with bulk density, a strong negative trend was found between soil organic matter (%) and bulk density (*R* ^2^ = 0.26, *P = *0.0921, *N = *12).

**Figure 6:**
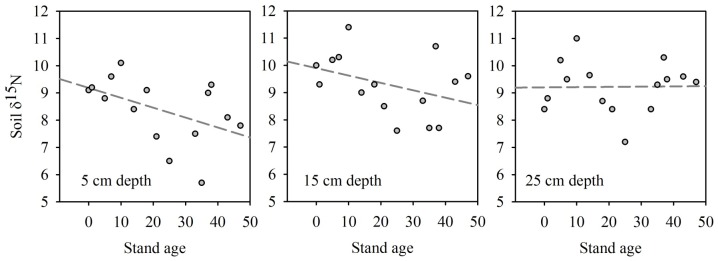
Soil δ^15^N values versus stand age by soil depth. A significant relationship exists between stand age and δ^15^N for all samples (*R* ^2^ = 0.26, *P* = 0.0881, *N* = 12) and a trend at 5 cm depth (*R* ^2^ = 0.26, *P* = 0.0881, *N* = 12).

The multiple regression model of soil δ^15^N which included soil depth was highly significant (*R* ^2^ = 0.23, *P* = 0.0285, *N* = 38), and showed a negative and positive relationship between soil δ^15^N and stand age (*t*-ratio = −2.07, *F*-ratio = 4.29, *P*-value = 0.0459) and depth (*t*-ratio = 2.25, *F*-ratio = 5.08, *P*-value = 0.0307), respectively, and no significant interaction (although see Figure 6 for trend). While the model of soil δ^13^C was also significant (*R* ^2^ = 0.28, *P* = 0.0111, *N* = 37), only a positive relationship between δ^13^C (which became less negative) and soil depth was found (*t*-ratio = 2.81, *F*-ratio = 7.90, *P*-value = 0.0083) - although a negative trend was found between soil δ^13^C (which became more negative) and stand age (*t*-ratio = −1.70, *F*-ratio = 2.90, *P*-value = 0.0983) which was significant when aggregated as described above. Soil δ^15^N within the secondary forest stands at mean 7.5, 15 and 25 cm depths were 8.25±1.26, 9.24±1.20, and 9.32±0.97 (‰), respectively, and soil δ^13^C values at these depths were −28.43±0.46, −28.14±0.96, and −27.50±0.99 (‰), respectively. However, recognizing the sample size constraints (*N* = 6), no pattern of increasing soil δ^15^N was found within the two primary forest stands (*P* = 0.3594) with soil δ^15^N of 9.15±0.07, 9.65±0.49, and 8.60±0.28 (‰) for the three depths, respectively, while soil δ^13^C did become significantly less negative with depth (*R* ^2^ = 0.83, *P* = 0.0079, *N* = 6; varying from −28.3 to −26.4 from 7.5 to 25 cm depth, respectively).

Soil PCA axes 1–3 explained 30%, 22%, and 15% of variation across all soil property variables, respectively (Table 2 soil axes). Soil PCA axis one was positively correlated with P1, P2, C, Mg, CEC, base cations, and sand content and negatively correlated with stable isotopes, and silt and clay content. As the strongest correlations were with the soil texture measures we refer to this axis as the soil texture axis. Soil PCA axis two was positively correlated with C, N, δ^13^C, K, MG, H, and negatively correlated with δ^15^N. As strong correlations were identified throughout this group, and in particular with soil C, we refer to this axis as the soil fertility axis [67]. While the texture axis had no significant relationship with stand age, the soil fertility axis had a significant positive linear relationship (PCA-2 = −2.73+0.10 * Stand age, *R* ^2^ = 0.42, *P*<0.0313, *N* = 11). Secondary forests, as compared to primary forests, had significantly lower soil δ^13^C (−28.20±0.53 vs. −27.25±0.21 (‰), respectively) and higher P2 (10.66±3.24 vs. 7.45±0.64, respectively).

### Leaf Traits

**Figure 7:**
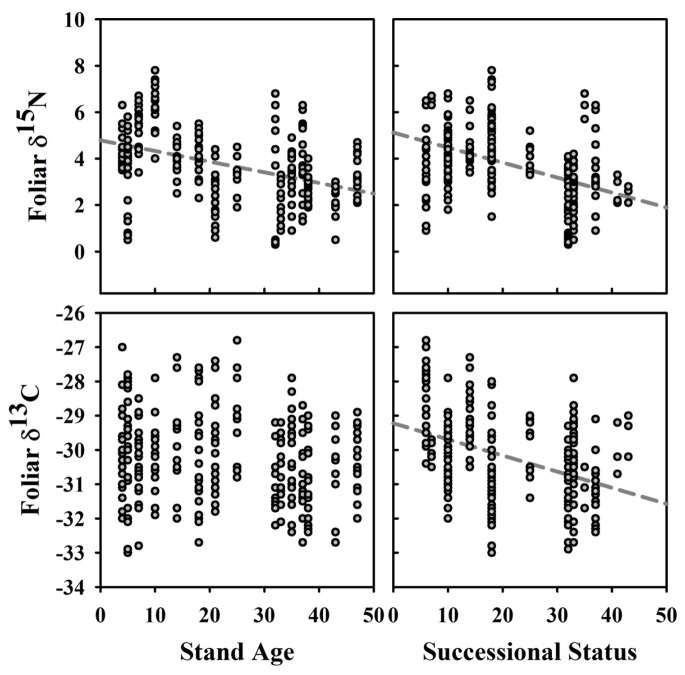
Foliar δ^15^N and δ^13^C values versus stand age and species successional status. Significant linear regressions are shown in grey.

**Figure 8:**
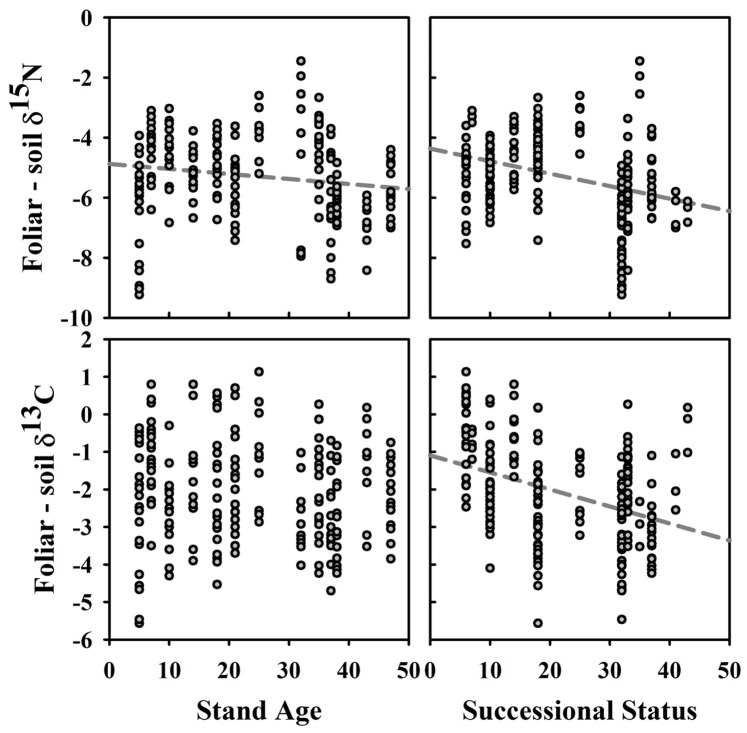
Foliar-minus-soil δ^15^N and δ^13^C values versus stand age and species successional status. Significant linear regressions are shown in grey.

The tree species, and their descriptive statistics, selected for inclusion in foliar analyses are provided in Table 4. While most leaf traits were significantly inter-correlated, foliar δ^15^N was only correlated, positively, with foliar %C and δ^13^C (Table 5). Prior to assessing leaf trait relationships with our six predictor variables, we used Pearson correlations to understand their relationships (Table 6). Stand age was significantly correlated with all of the predictor variables except for soil texture, which was only correlated with the stand structure axis. Species successional status was correlated with both the stand biomass and soil texture axes. The best subsets regression using these predictor variables showed that while stand age had two significant relationships with leaf traits, specifically positive with foliar δ^13^C (i.e., less negative) and negative with δ^15^N, species successional status had five significant relationships, with foliar N (+), %N (+), C:N (−), N:P (+) and δ^13^C (more negative) (Table 7 and Figure 7). Foliar δ^15^N had a significant positive relationship with soil δ^15^N (*R* ^2^ = 0.246, *P*<0.0001, *N = *232) whereas none was found between foliar and soil δ^13^C. A significant negative correlation, although weak, was found between Δδ^15^N_plant-soil_ and stand age (*R* ^2^ = 0.029, *P = *0.0089, *N = *232, but after exclusion of the youngest stand *R* ^2^ = 0.119, *P*<0.0001, *N = *204) and species successional status (*R* ^2^ = 0.118, *P*<0.0001, *N = *232), whereas a significant negative correlation was found only between Δδ^13^C_plant-soil_ and species successional status (*R* ^2^ = 0.149, *P*<0.0001, *N = *227) (Figure 8).

**Table 4:** Characteristics of tree species selected for analysis of foliar properties.

	Common name		Scientific name						
Species ID	Portuguese	Spanish	Genus	Species	Family	Wood density(kg m^−3^)	Successional age(years)	Total (sampled)stems**	Foliar n α
1	Ambaibo	Ambaibillo	Cecropia	polystachya	Cecropiaceae	280	6	76(14)	28 α
2	Favera	Serebo	Schizolobium	parahyba	Fabaceae	360	7	7(3)	6
3	Gameleira	Gomellera	Ficus	insipida	Moraceae	482	10	7(2)	4
4	Vela blanca		Miconia	sp.	Melastomataceae	587	10	128(22)	44 α
5	Ambaibo	Ambaibo	Cecropia	sciadophylla	Cecropiaceae	280	14	57(9)	18
6	Pente de Macaco	Peine de Mono	Apeiba	tibourbou	Tiliaceae	292	14	39(2)	4
7	Marupa	Chepereque	Jacaranda	cuspidifolia	Bignoniaceae	518	18	157(28)	56 α
8	Mutanba	Mutanba	Guazuma	crinita	Sterculiaceae	540	25	14(5)	10
9	Inga	Pacai	Inga	sp.	Fabaceae	603	32	86(20)	40 α
10	Urucuseco	Urucurana	Sloanea	sp.	Elaeocarpaceae	513	33	16(4)	8
11	Joao Mole	Joao Mole	Neea	sp.	Nyctaginaceae	454	33	36(11)	22
12	Louro preto	Laurel	Endlicheria	krukovii	Lauraceae	588	33	40(5)	10
13	Biorana	Trompillo	Pouteria	sp.	Sapotaceae	769	35	21(4)	8
14	Pama	Nui	Pseudolmedia	laevis	Moraceae	593	35	31(2)	4
15	Enviara caju	Piraquina	Onychopetalum	periquino	Annonaceae	619	37	27(8)	16
16	Guayabochi	Guayabochi	Capirona	decorticans	Rubiaceae	840	37	16(2)	4
17	Guariuba	Murure	Clarisia	racemosa	Moraceae	550	37	9(2)	4
18	Jachi	Palo santo	Tachigali	paniculata	Fabaceae	600	41	16(2)	4
19	Breu branco		Rollinia	calcarata	Annonaceae	520	43	16(2)	4
20	Balsamo	Balsamo	Myroxylon	balsamum	Fabaceae	796	43	3(2)	4

*Across all study sites, α = selected for individual species response analyses.

**Table 5:** Pearson correlations among leaf trait variables.

	P	N:P	C%	N%	C:N	δ^15^N	δ^13^C
N	0.66(264)***	0.24(263)***	0.12(260)*	0.85(262)***	−0.85(259)***	0.04(263)	−0.04(258)
P		−0.55(263)***	−0.28(259)***	0.61(261)***	−0.66(258)***	0.08(262)	0.33(258)***
N:P			0.49(258)***	0.14(260)*	−0.07(257)	−0.07(261)	−0.44(257)***
C%				0.15(259)*	−0.03(259)	0.25(259)***	−0.27(254)***
N%					−0.99(259)***	0.07(260)	−0.07(255)
C:N						−0.04(258)	0.03(253)
δ^15^N							0.20(258)**

Data is the correlation value (*N*) and *P*-value expressed as *<0.1, **<0.05, ***<0.01, and ****<0.001.

**Table 6:** Pearson correlations among the predictor variables stand age (years), species successional status (status), and the PCA derived axes of stand biomass and structure and soil texture and fertility.

	Status	Biomass	Structure	Texture	Fertility
Stand age	0.63(265)****	0.86(265)****	0.1(265)*	−0.01(223)	0.67(195)****
Status		0.54(265)****	0.02(265)	0.05(223)	0.46(195)****
Biomass			0.24(265)****	0.08(223)	0.39(195)****
Structure				0.64(223)****	0.01(195)
Texture					0.03(195)

Data is the correlation value (*N*) and *P*-value expressed as *<0.1, **<0.05, ***<0.01, and ****<0.001.

**Table 7:** Best subsets models of leaf traits versus stand age, species successional status, and Principal Component Analysis (PCA) derived gradients in stand biomass and structure, and soil texture and fertility.

			PCA Axes						
Leaf traits	Stand age	Species Successional Status	Biomass	Structure	Texture	Fertility	Adj-*R* ^2^	*P*-value	N
N	–	2.54 (6.46)**	–	–	–	–	0.021	0.0116	265
P	–	–	–	–	2.61 (6.83)***	–	0.026	0.0096	222
N:P	–	4.09 (16.76)^φ^	–	–	−3.79 (14.40)^φ^	–	0.115	<0.0001	221
C %	–	–	–	–	–	–	–	–	260
N %	–	2.40 (5.74)***	–	–	–	–	0.018	0.0173	262
C:N	–	−2.44 (5.97)***	–	–	–	–	0.019	0.0152	259
δ^13^C	3.58 (12.85)^φ^	−7.93 (62.86)^φ^	–	–	–	–	0.202	<0.0001	258
δ^15^N	−7.54 (56.92)^φ^	–	4.30 (18.50)^φ^	−3.17 (10.02)***	–	–	0.234	<0.0001	263

Data represents the *t*-ratio (*F*-ratio) and *P*-value significance, with increasing * representing *P*-values of 0.1, 0.05, 0.01, and 0.001, respectively, and φ <0.0001.

The stand biomass and structure axes had significant correlations with foliar δ^15^N - positive and negative, respectively - and soil texture had a significant positive correlation with foliar P and negative correlation with foliar N:P. Of all leaf traits only foliar %C was not significantly correlated with at least one predictor variable. For all leaf traits, species successional status had significantly higher explanatory power than stand age (Table 8). We then tested if correlations in leaf traits with stand age found across the species community also occurred within four individual species. No species had a significant relationship between foliar N, P, or N:P and stand age. Three species of four (*Inga* sp. being the exception) had significant negative relationships between foliar δ^15^N and stand age (*R* ^2^ ranged from 0.19–0.26) and two species (*Miconia* sp. and *J. cuspidifolia*) had significant positive relationships between foliar δ^13^C (less negative) and stand age (*R* ^2^ ranged from 0.09–0.13). Last, we tested for taxonomic and phylogenetic leaf trait signal. Although significant differences existed among species for all foliar variables (*R^2^* = 0.38 to 0.54, *P*<0.0001), significant phylogenetic signal (i.e., more than by chance alone) was found for only foliar %C (*K* = 1.2272, *P* = 0.0073). We do not make a case for phylogenetic trait conservatism or any evolutionary arguments, especially as our significant *K*-statistic value was only marginally larger than one.

**Table 8:** Regressions of foliar variables versus stand age (years)/species successional status (years).

Foliar variables	Combined	Stand age	Successional status
Linear regression models and model comparisons			
N:P	0.05 (8) 260***	0.01 (2) 261 NS	0.05 (15) 261***
		14***	1 NS
C:N	0.01 (2) 256 NS	0.00 (1) 257 NS	0.01 (4) 257*
		4	0 NS
δ^15^N	0.24 (43) 260****	0.18 (57) 261****	0.22 (74) 261****
		23****	9**
δ^13^C	0.20 (33) 258****	0.01 (3) 258 NS	0.17 (52) 258****
		31.3****	5.39*
Standardized slope values			
N:P	−0.57 NS/1.90***	NS	0.62
C:N	NS	NS	−1.65
δ^15^N	−0.33**/−0.52****	−0.66	−0.73
δ^13^C	0.02****/−0.07****	NS	−0.05

Statistics provided are Adj-*R* ^2^ (F) *df* and *P*-value. Model comparisons are conducted using the F-test in R and *P*-values are presented as: blank <0.1, *<0.05, **<0.01, ***<0.001, ****<0.0001.

## Discussion

We used a high-resolution forest succession chronosequence to evaluate potential predictors of leaf trait variation. We analyzed correlations among leaf traits and stand age, species successional status, and PCA-derived axes of stand biomass and structure and soil texture and fertility. While all leaf traits, with the exception of foliar %C, had significant correlations with at least one predictor variable, the strongest relationships were found between foliar stable isotopes and stand age and species successional status. Although our discussion focuses on leaf trait variation during secondary forest succession, we also discuss patterns identified in stand structure and composition and soil properties, focusing on variation in soil δ^13^C and δ^15^N.

### Stand Structure and Composition

The rapid decline in dead stems in the early stages of succession were likely a result of both remnant dead stems from the slash and burn cycle and rapid turnover in species composition during the first 10 years of succession. Areas having undergone multiple slash and burn cycles would be less likely to have remnant dead trees, however our plots had undergone only 2–3 years of agriculture prior to abandonment. Prach et al. (1993) [68] documented rapid turnover in species composition during the early stages of succession (<10 years) which, subsequently, slowed dramatically in a successional sere ecosystem. Similar changes in the rates of change in species composition and forest structure are highlighted by Chazdon et al. (2007) [141] for tropical forests. Our results indicated a similar progression with rapid species turnover, as shown through decreases in composition differences as compared to primary forest, beginning 15 years post-abandonment with the fastest rate of change (i.e., species turnover) during the first 10 years of forest succession (Figure 1), simultaneous with a reduction in the standard deviation of successional status. Such changes are in line with those predicted by the “Initial Floristic Composition” (IFC) hypotheses, which states that early successional stands will contain a large proportion of the species dominating in later stages of succession [69]. In our case, the rapid reduction in stand mean successional status in the early stages is due to high mortality of early pioneer species while the high variance in the early succession is due to the prevalence of later succession species (Figure 1). In older stands, pioneer species have died off due to differences in growth rates, longevity and shade-tolerance [70], reducing the variance of successional status in those stands.

Further insights are possible by separating overstory species from the community in general. The increase in community species richness was not seen in the overstory, similar to results found by Guariguata et al. (1997) [71] and Norden et al. (2009) [72], whereas slight increases in diversity were seen. This represents continual shifts in overstory composition, versus addition through succession. However, the increasing species richness differs from that postulated by the IFC hypotheses, as new species are continually added throughout succession and earliest stands having low richness and high dominance by a few short-lived pioneers. The species similarity changes are on par with those hypothesized by Oliver and Larson (1990) [25] and Waring and Running (2007) [24]. Although the understory approximated primary forest composition after only 50 years, the overstory remained completely different (Figure 4), similar to that found by Peña-Claros (2003) [23]. Waring and Running (2007) [24] state that primary forest composition will be attained between 100–400 years post-abandonment. However, it is not possible to simply extrapolate vertical growth rates of shade tolerant trees now dominant in the understory to predict composition changes in the overstory as many have negligible growth rates [73], with changes occurring in pulse events following disturbance related gap openings [74].

By 50 years, our secondary forests were similar in structure to our primary forest plots. Biomass was nearing the asymptote and average tree height and DBH had both begun to decrease. Similar dynamics have been seen during succession in the Amazon [23] and Panama [22], with the earlier stages of succession having the greatest rates of biomass accrual [75]. This relationship has been described as resulting from die-off of pioneer species simultaneous to establishment of long lived shade tolerant species, with more than 200 years being estimated to be required to attain primary forest biomass [76]. We found a quadratic relationship between stand age and the height of our emergent trees in which the mean height of emergent trees attained maximum values in intermediate stands. We postulated this to result from early successional species, with no recruitment in the understory, prior to species turnover to dominance by shade tolerant species [76], [77]. We found indications towards this occurring with the trees contributing the most to overstory composition in the intermediate aged stands to be early successional species growing past their identified species successional statuses (*R* ^2^ = 0.42, *P* = 0.0658, *N* = 13).

### Soil Properties

Changes in soil properties during secondary forest succession typically include increases in soil carbon [78]. Although the opposite was found by Schedlbauer and Kavanagh (2008) [45], in former pastures in Costa Rica, who explained that active non-crystalline clays and aluminum-humus linkages may have resulted in higher carbon stability during land use than that found in other studies. Other studies in the tropics have found pasture soils tending to have less carbon storage than cultivated soils [79], increasing extractable soil N and decreasing P stocks [80], and decreases in bulk density [81] (but see [82]), among other possibilities [9]. Generally, rapid decreases in total soil organic carbon (SOC) occur within the first 50 years following forest conversion, followed by slower losses until reaching a new lower equilibrium after 100 years. However, forest soils have significant memory and forest derived SOC represents >80% of the total pool if cultivation occurs for less than 5 years [83]. Global patterns of soil nitrogen are primarily controlled by mean annual precipitation and temperature that determine input from atmospheric deposition and N fixation, but during the slash-and-burn process, significant ecosystem nitrogen is lost through biomass removal, volatilization from combustion [84], and denitrification and leaching [85].

During stand development, we found significant increases in soil mass that, in part, resulted in increasing pools of carbon and nitrogen, but not phosphorus. In our case, increases in soil mass partly resulted from compaction following the slash-and-burn agricultural activity which reduced bulk density during the soil preparation, crop planting, and plant growth and harvesting, similar to that found by Brown and Lugo (1990) [187], contrary to that found following more intensive and more extended land uses such as pasture, such as found by Davidson et al. (2007) [37] in the top 0–10 cm depth profile, although when only considering this depth results from our study are similar. Feldpausch et al. (2004) [80] identified similar patterns during young successional stands (<14 years), with soil nitrogen increasing but phosphorus moving from below- to above-ground. Such dynamics are typical during succession as available soil phosphorus declines during nutrient redistribution from vegetation growth due to virtually no primary minerals remaining in the highly weathered soils [86]. Soil nitrogen, however, can increase through symbiotic nitrogen fixation, especially during early stages of succession [87], [88] and atmospheric deposition [89]. These changes were highlighted by the soil fertility axis, driven largely through N, increasing with stand age. Reductions in soil δ^15^N, although a non-significant negative trend with stand age, also formed an important contributor of the soil fertility axis.

Soil δ^15^N is determined by the equilibrium of δ^15^N inputs, outputs and internally through redistribution via plant uptake [55] and most soils have positive δ^15^N values due to accumulated losses [90]. In general, ecosystems with high rates of nitrogen fractionation resulting in soil ^15^N enrichment are viewed as having a leaky or open N cycle, typically with abundant N, versus ecosystems with a conservative or closed N cycle, and therefore reduced N loss, having reduced ^15^N enrichment [39], [91]. Input processes include: (a) soil ^15^N depletion through atmospheric inputs, including from precipitation [92], combustion [93], and nitrogen deposition [94]; and (b) soil ^15^N enrichment via symbiotic nitrogen fixation related fractionation during uptake [95]–[98]. Output processes include: (a) soil ^15^N enrichment through selective loss of ^14^N during decomposition related nitrification and denitrification [94], [99], including gaseous ^14^N losses to the atmosphere [100]; and (b) soil ^15^N enrichment through hydrologic leaching loss of ^15^N depleted nitrogen (relative to the soil) produced during nitrification, denitrification and ammonia volatilization [101]. Redistribution processes include: (a) soil ^15^N enrichment through discrimination against ^15^N during biological nitrogen fixation, or creation of ^15^N depleted compounds during decomposition and stabilization, and subsequent loss and/or assimilation of leachate from such activities at depth resulting in soil ^15^N depletion [98]; and (b) discrimination against ^15^N during plant uptake resulting in redistribution of depleted ^15^N from mineral soil at depth to plant biomass and eventually to the surface horizon [43].

Patterns of soil δ^15^N in depth profiles, now well established [99], [102], result from multiple factors, including: (a) the soil surface can become ^15^N depleted as litterfall accumulates at the soil surface while deeper soils can become ^15^N enriched due to increased mycorrhizal fungal activity [98]; (b) N loss from nitrification and denitrification can result in either: (i) a steady soil ^15^N enrichment with depth given abundant N availability [98]; or (ii) enriched soil ^15^N at intermediate depth in arbuscular mycorrhizal (AM) systems and/or sites of higher available nitrogen [103]; and/or (c) increases in soil fungi with depth resulting in soil ^15^N enrichment [104]. Differences in soil texture may also play a role in defining soil δ^15^N, with increasing clay % generally accompanying soil ^15^N enrichment [105], as well as land use intensity altering soil δ^15^N [106]. Of particular importance, burning alters the ^15^N pattern in soil profiles by eliminating the most ^15^N depleted organic layer [98] resulting in significant soil δ^15^N enrichment in the upper 20 cm [107].

In our secondary forest stands, soil δ^15^N both decreased with stand age and increased with soil depth. In the primary forest however, we found that soil δ^15^N was greatest at intermediate depth followed by decreases as would be expected for AM systems or those with higher N availability [103]. Differences along the stand age gradient were unlikely a result of soil clay content as no significant changes were found [105]. The pattern of soil ^15^N enrichment with depth may develop rapidly due to the large soil ^15^N enrichment in the upper 20 cm from combustion fractionation during the burning process [107], preferentially moving ^14^N downwards. Such dynamics would occur most rapidly during the agricultural phase and in early successional stands with less well developed root mats and reduced bulk density but continue throughout all stands. Although no significant interaction effect was found showing reduced profile significance with stand age, as would have been expected if our secondary forest ^15^N profiles were developing towards those found in the primary forests, this could take longer than our secondary stands studied [98].

The subsequent significant soil ^15^N depletion with stand age, which occurred most rapidly in the shallower depths most affected by burning (see trend in Figure 6), would then be due to simultaneous influence of: (a) atmospheric deposition of isotopically lighter ^14^N [94], of especial importance in this area due to the intense annual fire season [108]; (b) preferential leaching of soil ^14^N, and ^15^N depleted products, during the intense annual wet season [101], [109]; (c) changes in plant root distribution and increased foraging depth causing changes in the utilization of soil inorganic N pools [94] or acting as a biological pump to the surface via litterfall [183], of depleted ^15^N - although the soil ^15^N values found in deeper soils (>30 cm) were not measured in this study; (d) possible mycorrhizal fungal activity [98] – although such activities typically enrich soil ^15^N, and/or (e) mobilization of recalcitrant soil N to active pools [37], with recalcitrant pools being depleted in ^15^N if formed prior to the combustion event. Compton et al. (2007) [43] studied soil δ^15^N in secondary forests up to 115 years post agricultural abandonment in Rhode Island, USA and, with the exception of the organic horizon in which δ^15^N decreased, found ^15^N enrichment with stand age across all depths – although they found the same pattern of ^15^N enrichment with depth in the soil as identified in our study. These differences are likely due to very different land use intensities between the two studies, with the stands studied by Compton et al. (2007) [43] having undergone far greater land use intensity, including both mechanized agricultural and/or high intensity cattle grazing, prior to abandonment. Similar results – to Compton et al. (2007) [43] – obtained by Billings and Richter (2006) [44] may be also explained through the far greater mechanized agriculture prior to abandonment.

Variation in soil δ^13^C is generally thought to be a result of the dominant plant species composition, as decomposition related fractionation is small relative to that during carbon fixation [102], and is therefore driven by changes in the relative abundance of C_3_ (i.e., most plants) to C_4_ (i.e., corn, sugar cane, most tropical pasture grasses) plants [45]. Differences in foliar carbon isotope composition between C_3_ and C_4_ species reflect differences in their photosynthetic pathways, with C_3_ species have a δ^13^C range of −32 to −20% whereas C_4_ species range from −17% to −9% [110]. However, soil ^13^C enrichment also occurs during auto- and heterotrophic soil CO_2_ efflux - which is ^13^C depleted - from decomposition of surface litter [111], and during oxidation of soil organic matter and soil humification processes [112]–[114], or possibly depletion from root respiration [115]. The isotopic composition of autotrophic respiration (i.e., leaves, twigs, roots) is most likely derived from young newly fixed carbon, with the related δ^13^C value, whereas heterotrophic respiration (i.e., decomposition) will likely have a different isotopic signature depending on the carbon turnover rate of the labile available carbon pools [114], [116]. Such differences have been identified in soils throughout the tropics [117], [118], with a δ^13^C of −25% commonly used to represent the value of the stable pool [119]. Profiles showing increasing δ^13^C with depth, which occur independent of soil type [120], are commonly found under conditions of stable vegetation cover and low soil disturbance (i.e., tilling) [121] and result from reduced decomposition related isotope fractionation with depth [112] and decreases in the size of soil organic matter fractions [122]. In these situations, surface carbon is generally of young origin and labile with increasingly old and recalcitrant SOM pools with depth [119].

Changes in soil δ^13^C during forest succession reflect: (a) variation in the turnover rates of soil organic matter (SOM), including decreased stability of some previously stable – potentially ^13^C enriched - C pools following burning [119] – including changes in relative contribution of microbial vs. plant soil organic matter [114]; (b) changes in vegetation, including shifts between C_3_ and C_4_ species, as soil ^13^C composition greatly reflects that of the dominant vegetation [102], due to low rates of fractionation during decomposition relative to fixation [123] – although Billings and Richter (2006) [44] found very slow incorporation of new plant carbon into soil horizons beyond the uppermost (<10 cm) layers; and (c) a large flux of depleted ^13^C plant lignin and root organic matter, or remnant charcoal, from combustion during the deforestation process [119], [181] – which can result in confusion as pasture carbon isotope composition could appear similar to intact forests. Decreased soil δ^13^C in abandoned pastures and agricultural areas, as compared to forest, has been well established [45], [83] with increasing historical years of cultivation of C^4^ species having a positive (less negative) relationship with soil δ^13^C values (forest soil δ^13^C = −24, vs. 17 and 60 years of cultivation = −23 and −16, respectively; [83]).

Although the soil ^13^C depletion with stand age in our study contrasted that found by Billings and Richter (2006) [44] whose stands were regrowing on sites subjected to intensive agriculture of cotton (a C_3_ species), it was similar to that found by Lopez-Ulloa et al. (2005) [124] who studied forest stands regrowing on sites opened through slash-burn and used as pasture. In our study, changes in soil δ^13^C were unlikely dominated by a C_4_ species signal related to the agricultural (or pasture) phase as identified in studies following more intense land use [44]. However, although subsistence agricultural species grown in our study area were primarily C_3_, including bananas, beans, rice and yucca, the dominant crops included corn, a C_4_ species, and rice. More likely, the decrease in soil δ^13^C seen with increasing stand age represents a gradual return to stable primary forest values following: (a) a large signal from burning related ^13^C enrichment during the slash-and-burn process [119], (b) a smaller ^13^C enrichment signal from the corn, a C_4_ species, cultivated during the 1–2 year no till agriculture period [110], (c) a transition to C_3_ species dominated forest composition with more stable disturbance dynamics, and (d) continual input of ^13^C depleted carbon during the dry season from wildfires [125]. Soil δ^13^C in our study did increase with depth as expected given reduced rates of decomposition; with the development of a linear relationship between ^13^C enrichment and increasing depth occurring in the primary stands as expected given their more stable forest structure and plant composition [121]. Our primary forest soil δ^13^C were considerably more enriched than those in our oldest secondary forest stands, possibly indicating long term ^13^C enrichment via leaching.

### Leaf Traits

At a global scale leaf traits have been found to represent a continuous gradient dubbed the ‘leaf economic spectrum’, ranging from short-lived high photosynthetic capacity leaves to long-lived, thick, low photosynthetic capacity leaves [126]. However, further analyses have revealed that the range within groups along this gradient is often larger than the differences among them [127] – in part related to broad changes in mean annual temperature and precipitation – and shifts from long-lived low nutrient leaves in low fertility soils to high nutrient content leaves on more fertile sites [128]. Leaf trait variation within a primary forest (i.e., a relatively stable environment) is further constrained by phylogenetic, taxonomic or functional group differences [28], [129]–[132], growth environment [133]–[135] and propagation strategy [27], among many factors [8]. Understanding variation in leaf traits through forest succession (i.e., a highly dynamic environment) integrates across these groups as it moves though variation in inter- and intra-species competition, differences in plant growth and reproductive strategy, and plant-soil feedbacks [23], [136], [137].

During secondary forest succession, as well as over longer term ecosystem succession processes [175], [176], foliar trait dynamics may provide information unique from that shown in the stand or soil properties, or largely result from overall stand changes or differences in soil properties. Understanding these factors is critical to enable the use of leaf traits, including nutrient concentrations and stable isotope ratios, to understand successional dynamics. We start by investigating, for each measured leaf trait, possible phylogenetic control over leaf trait variation, then focus on how changes in stand structure and soil properties may influence their expression. In addition, we focus on situations where leaf traits may follow different trends than those occurring belowground and thereby provide additional information.

First, we investigate whether differences in leaf traits are a result of phylogenetic control (Figure 2). Powers and Tifflin (2010) [130] found that inter-specific variation accounted for 57–83% of leaf trait variance across 87 tropical dry forest tree species whereas He et al. (2010) [129] found that 27% of leaf trait variation was due to phylogenetic differences in a grassland ecosystem. These contrast with our results, which showed phylogenetic signal only in leaf %C. However, our results strongly agree with those of Fyllas et al. (2009) [131] and Asner and Martin (2011) [138], indicating that that phylogeny accounts for 50–80% of the variation in foliar %C, depending upon site characteristics. As such our results can be explained by considering our study system which represents a large gradient of stand structure and species composition, in which the stand age or species successional status gradients outweigh differences among species. In any individual stand however it is entirely plausible that inter-species differences are of primary importance to explain leaf trait variation, although Letcher (2010) [139] found significant over-dispersion of species - across the phylogenetic community - at stand scale during forest succession as a result of rapid transition through species ‘functional’ groups (i.e., along the species successional status gradient). In particular, Letcher (2010) [139] found that no phylogenetic structure existed in the youngest stands potentially resulting from the rapid rate of change in species, as indicated in Figure 1 for mean stand species successional status. Fyllas et al. (2009) [131] analyzed leaf traits from 508 species distributed across a range of soil types and precipitation regimes and found that foliar %C (as identified in our study), %N and Mg concentration were highly taxonomical constrained. However, foliar P, K, Ca and δ^13^C were influenced by site growing conditions, with soil fertility being the most important predictor for all variables. Mean annual temperature (MAT) was negatively related to foliar N, P and K, and MAP was positively related to foliar %C and δ^13^C. Townsend et al. (2007) [36] used foliar N:P ratios to show that differences in growth latitude or mean annual precipitation (MAP) had a non-significant influence on foliar N:P ratios within the tropics, while large significant differences were found among species, between the dry and wet season, and with soil order. Pringle et al. (2010) [140] found no phylogenetic signal among the leaf traits of trees growing in a seasonally dry tropical forest in Mexico, which they explained as indicating that selective pressures (i.e., functional convergence) constrained leaf traits.

In the context of forest succession, phylogenetic signal over all successional stands may be less relevant to understanding temporal dynamics than how species functional qualities change through time [8], [28]. In particular, given successional theory, it is more likely that changes in a suite of functional characteristics are occurring through succession [142], in part due to environmental niche partitioning and strategy differentiation [182]. The importance in functional differentiation during succession was supported by the lack of phylogenetic signal across the species successional status gradient of our studied trees, although further analyses are required to unravel phylogenetic – successional status interactions during succession. Huc et al. (1994) [143] studied differences in leaf traits among categorical divisions of species successional status in the French Guiana. Wood cellulose δ^13^C, leaf gas exchange and leaf water potential were shown to differ significantly between pioneer and late stage successional guild tree species growing in a common garden [143], and the authors highlighted the need for additional research on successional guild control over ecophysiological function [29].

Given significant changes in soil N and P pools, as expected given successional theory predicting changes from N to P limitation during forest succession [144], we anticipated a corresponding positive shift in the foliar N:P ratio. Our results show, that in our study area, changes in foliar N:P are related to changes in species successional status (Table 7). Although stands younger than 10 years of age do have stand average foliar N:P value less than 14–16, the N (lower) to P (higher) limitation threshold described by Townsend et al. (2007) [36] in a literature review, most are above this threshold indicating that significant N limitation is not occurring in our study area. This was expected given the large N pulse seen in the soil δ^15^N of younger stands, while N in older stands would have accumulated due to biological N fixation and atmospheric deposition. Although foliar N varied with successional status, foliar P varied only with the soil texture gradient, with foliar P tracking changes in soil P. Soil P however did not vary with stand age, indicating these changes are unrelated to successional process, but instead had a significant negative correlation with soil clay. These results are similar to those identified by Silver et al. (2000) [75] who found that when soil clay content increased, labile P decreased while total P increased, due to P being easily complexed with exchangeable Al and Fe. Our soil P analyses (weak Bray) were designed to assess the labile P pools [145], [146], which would have the greatest effect on foliar P concentrations. Therefore, in this study, our results indicate that soil P represents variation in soil clay content resulting from differences in our chronosequence not related to successional processes, which was then mirrored in the foliar P content. Changes in foliar N however resulted from, to a much lesser extent, increasing N availability during stand development, and primarily from shifts in species successional growth strategies, indicative of species growing on nutrient rich sites [128], and similar to results from Yan et al. (2006) [142] showing increasing foliar N among three tree species increasing in successional status which was attributed to changes from conservative to resource spending in later successional species due to increases in soil fertility. Our data shows this pattern to occur across both the entire species community as well as within individual species.

While foliar δ^15^N reflects in large part the isotopic composition of the available soil nitrogen pool [55] – which in turn results from species composition [190] and N cycle openness [91], various sources of fractionation exist which can provide additional information over soil δ^15^N alone, e.g., biological nitrogen fixation [147] discriminates against ^15^N during soil N to plant transfer decreasing foliar δ^15^N. During forest succession, theory predicts decreasing N limitation and therefore greater openness of the N cycle, resulting in increased soil ^15^N fractionation and therefore ^15^N enrichment. Contrary to this however, simultaneous reductions in foliar and soil δ^15^N during succession have been found in several studies. Wang et al. (2007) [148] attributed decreases in soil and foliar δ^15^N on an old field in northern Virginia following intensive agriculture because of increases in woody ectomycorrhizal (EM) and herbaceous vesicular-arbuscular mycorrhizal (VAM) – also referred to as arbuscular mycorrhizal (AM) due to vesicles not being produced by all fungi - hosted fungi causing discrimination against soil ^15^N during uptake. They highlight that VAM abundance increases into early successional stands and then reduces in intermediate aged stands when EM hosted fungi (which discriminate more against ^15^N than VAM) increase [149], [150]. In addition to finding community level dynamics of decreasing foliar δ^15^N they also identified decreasing patterns among individual species, similar to our findings. They conclude that soil δ^15^N is the major factor driving foliar δ^15^N due to a highly significant positive relationship (R^2^>0.90). In our study, we likewise found a highly significant relationship, although the R^2^ value of 0.25 indicated that other processes might be constraining foliar δ^15^N dynamics, such as root development, which likely provides access to increasingly depleted N at depth [43].

Compton et al. (2007) [43] use the divergence between foliar and soil δ^15^N values (Δδ^15^N_plant-soil_) to address drivers of foliar δ^15^N variation, including rooting depth, as it allows for direct comparison of N fractionation during plant uptake, with increasing values indicating increased fractionation. Although we perform a similar study of divergence, we recognize that our soil analyses were limited to <30 cm whereas rooting depths are well distributed up to 2 meters depth [151], and sometimes extend much deeper (>8 meters in some primary forests in the Amazon; [152]). Divergence results, in part, from: (a) differences in the rate and abundance of EM and AM activity [184]; (b) changes in plant efficiency of mineral N cycling during succession, as has been found for increasing water availability [101]; and (c) changes in plant uptake between organic/NH_4_^+^ (cool temperate) and N0_3_^−^ (tropical) [55], although climatic differences would be minimal among our co-located study stands.

As mycorrhizal activity results in depletion of foliar ^15^N we expected an increasing Δδ^15^N_plant-soil_ divergence during forest succession. This trend would be most pronounced if soil δ^15^N were being measured at the same depths that mycorrhizal activity was greatest, and diluted if soil from shallower depths having substantial litter input and therefore soil ^15^N depletion was used. During forest succession, simultaneous increases in rooting depth – as we collected leaves from top-of-canopy positions - would influence this relationship as we found soil ^15^N enrichment with depth, but further detailed analysis of soil mycorrhizal activity and soil δ^15^N to depths of at least 3–5 meters would be required to interpret possible interactions. While stand age and species successional status both exhibited the significant increases in divergence as indicative of increased mycorrhizal activity, species successional status was a much stronger predictor. Although we do not have data regarding specific mycorrhizal relationships among our study species, an increasing probability of mycorrhizal associations, and in particular AM – versus EM - associations which the vast majority of tropical tree species have [185], [186], occurring in later successional status species could explain this correlation – thus changes in species successional status during stand age would drive Δδ^15^N_plant-soil_ divergence depletion via an iterative feedback loop, simultaneous to a general trend of ^15^N depletion occurring across the species community as well as within individual species through stand age which indicates that ecological processes other than species successional status transitions are driving overall ecosystem δ^15^N depletion patterns – as discussed previously above – but such transitions may be accelerating ongoing ^15^N depletion patterns and in particular at shallower soil depths incorporating increasingly more depleted ^15^N via organic matter input.

Although patterns of decreasing δ^15^N were found by our study, the driving factors likely differ in our study from those of Compton et al. (2007) [43] and Wang et al. (2007) [148]. The successional chronosequence used in these studies did not undergo a burning cycle directly prior to abandonment, which significantly enriches soil ^15^N, rather they propose that their decreases resulted dominantly from: (a) shifts in rooting depth; (b) fractionation during plant uptake, (c) increased mycorrhizal activity (although no N-fixers were present in the Compton et al. (2007) [43] study site), and (d) changes in soil N cycling rates. Processes dominating our study N dynamics were likely more similar to those Davidson et al. (2007) [12] who found that foliar N, N:P, and δ^15^N all increased during forest succession. Likewise, we found increases in foliar N:P, driven by increases in N but no change in P, but conversely found decreasing foliar δ^15^N. We propose that although similar dynamics of N cycle recuperation (i.e., increasing leakiness and therefore N fractionation with stand development) are occurring within our study area, changes in foliar δ^15^N are more representative of a decrease in soil δ^15^N following high initial enrichment from combustion during the slash-and-burn process. The study plots selected by Davidson et al. (2007) [37], although likely burned at various points, had existed long enough in an agricultural or pasture state to stabilize at more depleted δ^15^N values prior to abandonment, with increasing time in pasture shown to result in increasingly depleted soil δ^15^N values [99].

Variation in foliar δ^13^C results primarily from differences in the ratio of internal (c_i_) to atmospheric (c_a_) CO_2_ concentrations, in which a decreased internal CO_2_ concentration relative to atmospheric (i.e., via closed stomata or rapid photosynthesis) results in decreased ^13^C discrimination and enrichment (less negative) foliar δ^13^C values [153]. In C_3_ plants, foliar ^13^C discrimination occurs primarily during photosynthetic CO_2_ uptake by ribulose1,5-bisphosphate (R_u_P_2_) carboxylase [154] and during gaseous diffusion through the boundary layer and leaf stomata [155], and is moderated by the CO_2_ diffusion rate into the leaf and rate of carboxylation [155]. Therefore photosynthesis has a depleted ^13^C uptake and tends to enrich the surrounding atmosphere while respiration has a depleted release and tends to deplete the atmosphere in ^13^C [156]. Factors affecting the c_i_/c_a_ ratio include: (a) variation in the concentration of source CO_2_ [153]. However, differences in atmospheric CO_2_ are likely minor and not relevant in our study, and respiration derived CO_2_, which is ^13^C depleted (δ^13^C∼−28% [157]) relative to atmospheric (and respiration itself causes little fractionation [158]), is not significantly different when leaves are sampled in locations of unimpeded air circulation [42] or greater than 7 m above ground in an intact primary tropical forest [157], [159]; (b) C_3_ or C_4_ photosynthesis [110], which result in typical foliar δ^13^C values of approximately −25% and −12%, respectively [110]; (c) a wide variety of environmental stresses which may result in alter the ratio of conductance to photosynthesis [160], such as soil drought which influences conductance more than photosynthesis [161], [162], with increasing drought resulting in enriched (less negative) foliar δ^13^C values; (d) differences in internal gas exchange in thick leaves, such as found by Vitousek et al. (1990) [42] where enriched (less negative) foliar δ^13^C (ranging from approximately −29.5 to −24.5 from low to higher elevations, respectively) was found within *Metrosideros polymorpha* at higher elevations as a result of increasing internal resistance to CO_2_ diffusion to sites of carboxylation within the leaf, associated with increased leaf mass per area (LMA), as well as thicker mesophylls and increased %N [163]; (e) differences in time-integrated water use efficiency (WUE) – assessed as the ratio of leaf assimilation rate of CO_2_ (A) to leaf water vapor conductance (g) – with increasing WUE (i.e., decreased water vapor conductance relative to photosynthetic capacity) resulting in enriched (less negative) foliar δ^13^C [153]; and (f) differences in growth location, such as understory environments with decreased irradiance (low A) and higher relative humidity (open stomata) having greater c_i_/c_a_ ratio resulting in depleted (more negative) foliar δ^13^C values [155].

Differences in foliar δ^13^C, resulting from combinations of the above mentioned factors, may vary in relation to: (a) species differences; (b) spatial location; and/or (c) disturbance history. Bonal et al. (2007) [47] investigated foliar physiology of seedlings of species belonging to different successional groups, in a greenhouse experiment on seedlings, and found that although pioneer (fast growing early successional) species had higher assimilation rates, reduced water use efficiency resulted in depleted (more negative) foliar δ^13^C values than fast-growing late successional species. Interestingly, they found no linear pattern of foliar δ^13^C change, but rather a V pattern, suggesting a transition among factors driving discrimination, and highlighted the need to study foliar δ^13^C dynamics along more complete successional gradients. Such non-linear variation might result from differences in seedling growth strategies, with: (a) low WUE resulting in ^13^C depletion (greater discrimination) in pioneer species; (b) high A but higher WUE resulting in relative ^13^C enrichment in intermediate successional species; and (c) very low rates of C0_2_ assimilation offsetting high WUE causing ^13^C depletion in late successional species. Huc et al. (1994) [143] had similar findings using δ^13^C of cellulose in wood cores from adult trees growing in artificial stands of pioneer and late stage forest species. They found that pioneer species had the greatest ^13^C discrimination, resulting from: (a) lower WUE; (b) high maximum conductance values; and (c) high specific hydraulic conductance, indicating an increased competitive ability for water and nutrient uptake in pioneer species.

Variation in foliar LMA and δ^13^C often follow predictable spatial patterns within forests. Domingues et al. (2005) [164] found LMA to increase and foliar δ^13^C to become enriched (less negative) with increasing height in an Amazonia old growth forest as a result of increased A values (due to increased irradiance) resulting in reduced c_i_ – increased c_i_/c_a_ ratio. Ometto et al. (2002) [159] had similar findings in the Brazilian Amazon, which they attributed to gradients in light and humidity – and specifically not to variation in source CO_2_ isotope composition, which varied by only 3% throughout the full canopy profile - affecting the ratio of leaf photosynthetic capacity and stomatal conductance. However, light gradients do not necessarily result in variation in carbon isotope discrimination as A and g typically change simultaneously keeping c_i_/c_a_, and therefore foliar ^13^C fractionation, relatively constant [188] – at least within an individual species with low leaf plasticity, as leaves growing under increased light availability can have higher LMA values [42]. Additional variation may also result from increasing tree height with succession, as height increases have been found to result in ^13^C enrichment due to reduced conductance and increases in LMA [165] – although this pattern is non-linear and most pronounced above 30 meters which is greater than the maximum tree height in all our secondary forest stands. Although not the subject of their analyses, variation in height within a forest may occur simultaneous with changes in species composition resulting in taxonomically constrained variation in foliar δ^13^C.

Foliar δ^13^C variation also occurs within individual tree crowns. Waring and Silvester (1994) [166] found that the aspect of exposure and branch length accounted for 6% of the foliar δ^13^C variation within individual crowns of *Pinus radiata*, with shorter branches or shaded aspect of exposure being related to enriched (less negative) foliar δ^13^C values. These differences resulted from decreased stomatal conductance on the longer branches. Differences in foliar δ^13^C among study sites typically results from a combination of the factors identified above. For example, Ehleringer et al. (1986) [167] found a clear decrease in foliar δ^13^C from disturbed to undisturbed sites, indicating decreased CO_2_ concentrations and increased water use efficiency (WUE). They explain this difference however through large changes in irradiance as altered through leaf canopy position and overstory density – likely causing changes in LMA.

We identified a highly significant decrease in foliar δ^13^C with increasing species successional status, but not with increasing stand age. Many of the factors identified above are: (a) related to either stand structural or soil properties, which would change with stand age and therefore result in no linear pattern; or (b) were normalized for across our study stands, such as topographic differences, elevation or climatic conditions. As all our leaves were collected from top of canopy full sunlight and atmospherically well circulated positions, differences in source CO_2_ or illumination are unlikely to have had a significant impact. Increases in internal CO_2_ diffusion resistance, as identified by Vitousek et al. (1990) [42], are also unlikely play a major role, as early successional species (and most others as well) have, relative to high elevation *Metrosideros polymorpha*, thin leaves. In addition, internal diffuse resistance would result in reduced discrimination (enriched foliar δ^13^C) in the later successional species having greater LMA – the opposite to the positive relationship between increasing ^13^C depletion with species successional status we found in our analyses.

Given the significant relationship with species successional status, but not stand age, described above, variation in foliar δ^13^C appears to be almost entirely related to species differences in leaf physiology and growth strategy correlated to their species successional status. While we did not collect data on LMA for our study species, Poorter et al. (2004) [32] quantified leaf traits of <2 m tall saplings across a successional gradient in the Bolivia Amazon and found significant decreases in specific leaf area (converse of LMA), nitrogen, water content and increases in C:N and lignin in later stage successional species. If similar patterns in LMA exist across adult individuals, as was found by Reich et al. (1995) [29] in the Venezuelan Amazon where later stage successional species had increasingly lower rates of photosynthesis, conductance and SLA, and increased leaf life span and toughness, then decreasing conductance relative to A could result in depleted (more negative) foliar δ^13^C values. The strong negative relationship between foliar δ^13^C and species successional status, but not stand age, further emphasizes that this trend is occurring simultaneous to – but independent of - the negative relationship shown between soil δ^13^C and stand age, which is in large part driven through changes in the foliar δ^13^C input during transition through species succession status. Given the non-significant relationship between stand age and foliar δ^13^C, the significant increase in Δδ^13^C _plant-soil_ we identified with increasing stand age indicates that either: (a) the incorporation of the foliar δ^13^C into the soil slows with increasing stand age; (b) that a significant source of soil δ^13^C enrichment develops with increasing stand age; or (c) a significant source of soil δ^13^C diminishes with stand age. Changes in foliar δ^13^C incorporation into the soil are directly related to the rapid increases in the quantity of decomposers during early stand development which can cause an exponential increase in the rate of overall decomposition, as the decomposers have access to abundant high quality litterfall from fast growing pioneer species, followed by reduced but stable rates of decomposition later in succession of the lower quality litterfall from slower growing tree species [31], [168]. However, increasing soil ^13^C enrichment via auto- and hetero-trophic respiration and microbial fractionation during decomposition could serve to offset soil ^13^C depletion resulting from: (a) the loss of the initial large ^13^C enrichment pulse following the burning; (b) incorporation of increasingly depleted ^13^C organic matter into the soil; and (c) transition to all C^3^ species, and thereby causing the increasingly negative Δδ^13^C _plant-soil_ relationship.

## Integration and Conclusion

During forest succession competition among species for resources, including light, water and soil nutrients, play an important role in species composition [169]. Considering variable resources, alterations in composition during forest succession are likely partially explained through resource niche partitioning [170]. Tree species from differing successional guilds may express different competitive capacities or nutrient use strategies during stand development [171], including shifts in nutrient use strategies from ‘conservative consumption’ to ‘nutrient spending’ – although this is most related to shifts in plant functional type rather than community-scale dynamics [142]. The interactions between these factors, including differences among taxonomic and physiological and functional diversity, has been described as being one of the least understood themes in tropical forest ecology [28], [172]. It is difficult to separate soil and foliar processes as they develop through complex feedbacks [136]. For example, changing successional vegetation plays a crucial role in making nutrients from the total soil pool available to plants [82] which feeds back to influence which species are best adapted to succeed and their spatial distribution [189]. Processes, such as the introduction of exotic species [173], can alter dynamics - similar to the continuous dynamic of feedback restructuring that occurs during secondary succession. Successional dynamics and biomass accumulation following abandonment depend in large part on the initial disturbance [174].

In this study, we found soil N to increase during forest succession while P remained stable. This was expected given the increasing influence of biological nitrogen fixation during succession and progressive depletion or incorporation of P into aboveground biomass. Increasing foliar %N values were shown with increasing species successional status indicating changes in growth and competitive strategy under conditions of varying N availability. Foliar P however was related to soil texture, not soil P concentration, and therefore represented differences among the plots not related to forest succession. The significant soil δ^15^N depletion with stand age likely does not represent a progressive tightening of the nitrogen cycle; rather it represents the balance of other fractionation related nitrogen cycling processes in a post-fire induced ^15^N enrichment context. While foliar δ^15^N is largely determined by the soil isotope composition, increasing divergence with species successional status indicates an increase in nitrogen isotope fractionation in older stands by later succession species, possibly as result of increased mycorrhizal activity and changes in root foraging depth. Soil δ^13^C is however in large part determined by the plant composition, with few plant-soil carbon isotope feedbacks, that is again partly determined by the initial burning related soil ^13^C enrichment.

Foliar δ^13^C depletion with increasing succession status, but not stand age, highlights the changes in tree growth and competition strategy during succession from thin fast growing leaves with rapid A and low WUE to slow growing species with thick well protected long lasting leaves having reduced rates of A but increased WUE. Increasing divergence between soil and foliar δ^13^C may be a result of increases in the quantity - but less likely the rate – of carbon cycling processes, such as decomposition, as stands age. Foliar %C was unrelated to stand age or species successional status but had significant phylogenetic signal, which was not found across the species successional status gradient. Phylogenetic constraints on foliar %C have been identified in other studies in less dynamic study systems, and likely other leaf traits in our study would exhibit phylogenetic signal under more stable (i.e., primary forest) conditions. The interplay among soil and foliar factors, with more specific quantification of the competitive processes during forest succession, requires further investigation and will be the focus of future work.

## Supporting Information

File S1 **Stand, tree, soil and foliar datasheets used in this study.**
(ZIP)
